# Conditional knockout of ITGB4 in bronchial epithelial cells directs bronchopulmonary dysplasia

**DOI:** 10.1111/jcmm.17948

**Published:** 2023-09-12

**Authors:** Yu Chen, Wang Jiang, Jin‐Mei Wang, Xiao‐Di Ma, Di Wu, Le‐Xin Liu, Ming Ji, Xiang‐Ping Qu, Chi Liu, Hui‐Jun Liu, Xiao‐Qun Qin, Yang Xiang

**Affiliations:** ^1^ School of Basic Medicine Central South University Changsha China; ^2^ Department of Medical Laboratory, School of Medicine Hunan Normal University Changsha China; ^3^ School of Medicine Foshan University Foshan China

**Keywords:** BPD, FAK, GSK3β, ITGB4, SOX2

## Abstract

Neonatal respiratory system disease is closely associated with embryonic lung development. Our group found that integrin β4 (ITGB4) is downregulated in the airway epithelium of asthma patients. Asthma is the most common chronic respiratory illness in childhood. Therefore, we suspect whether the deletion of ITGB4 would affect fetal lung development. In this study, we characterized the role of ITGB4 deficiency in bronchopulmonary dysplasia (BPD). ITGB4 was conditionally knocked out in CCSP‐rtTA, Tet‐O‐Cre and ITGB4^f/f^ triple transgenic mice. Lung tissues at different developmental stages were collected for experimental detection and transcriptome sequencing. The effects of ITGB4 deficiency on lung branching morphogenesis were observed by fetal mouse lung explant culture. Deleting ITGB4 from the airway epithelial cells results in enlargement of alveolar airspaces, inhibition of branching, the abnormal structure of epithelium cells and the impairment of cilia growth during lung development. Scanning electron microscopy showed that the airway epithelial cilia of the β4^ccsp.cre^ group appear to be sparse, shortened and lodging. Lung‐development‐relevant factors such as SftpC and SOX2 significantly decreased both mRNA and protein levels. KEGG pathway analysis indicated that multiple ontogenesis‐regulating‐relevant pathways converge to FAK. Accordingly, ITGB4 deletion decreased phospho‐FAK, phospho‐GSK3β and SOX2 levels, and the correspondingly contrary consequence was detected after treatment with GSK3β agonist (wortmannin). Airway branching defect of β4^ccsp.cre^ mice lung explants was also partly recovered after wortmannin treatment. Airway epithelial‐specific deletion of ITGB4 contributes to lung developmental defect, which could be achieved through the FAK/GSK3β/SOX2 signal pathway.

## 1 INTRODUCTION

The abnormal intrauterine development of the fetal lungs is closely related to the serious diseases of the neonatal respiratory system, such as BPD. Increasing evidence shows that the long‐term effects of fetal lung development impairment can last till adolescence, even adulthood. Compared with full‐term newborns, newborns with BPD are susceptible to airway hyper‐responsiveness and face a higher incidence of respiratory diseases such as asthma and chronic obstructive pulmonary disease (COPD) in their adulthood.^1^, ^2^ At present, the aetiology and the pathogenesis of BPD are still unclear. The immature lung development and lung damage caused by multiple impairments, based on genetic susceptibility, in utero and after birth, were generally considered BPD's essence.^3^

Our previous studies have found that the expression of integrin β4 (ITGB4) is downregulated in asthma patients. The sequencing of the promoter region of β4 integrin sampling from asthma patients testified that high‐frequency base mutation exists in the fixed site among the region and shows corresponding relation to asthma susceptibility.^4^ Foregoing results suggest that the deficiency of ITGB4 could be relevant to asthma susceptibility. Although the pathogenesis of asthma has not been fully elaborated, its high incidence in children implies a certain connection between lung development and asthma susceptibility.^5^ Based on the foresaid facts, we speculated that the expression defect of ITGB4 may affect fetal lung development and has a continuous effect till adolescence or even adulthood, thereby increasing the susceptibility of chronic respiratory system diseases such as asthma.

According to our research, ITGB4 deficiency led to mucus hypersecretion and MUC5AC overexpression in the small airway of RSV‐infected juvenile mice.^6^ Decreased ITGB4 expression results in increased lung tissue stiffness and impairs the adaptation of bronchial epithelial cells to substrate stiffness.^7^ Besides, research on the role of integrins in multi‐organ development was published; integrins α5 and β1 were involved in the development of the cardiovascular system, among which integrin β1 also plays an essential role in oral epithelium development. Furthermore, the lack of integrin α8β1 disrupted kidney organogenesis by disrupting the epithelial–mesenchymal interactions.^8^, ^9^, ^10^ As to pulmonary development, it has been documented that the deficiency of integrin α3 or β1 in mice manifested as bronchial branching defects and impaired epithelial cell differentiation and maturation.^11^, ^12^ Thus, it is reasonable for us to infer that ITGB4 might have a vital impact on lung development.

With its impact on the morphology, polarity and direction of movement of the cells, pulmonary matrix has a decisive effect on fetal lung development process, including the growth of lung bud, branching, bronchi forming and alveolarization.^13^, ^14^, ^15^ Integrins are widely distributed in the cell membrane of various organs. The combination of integrins with extracellular matrix leads to the reconstruction of the actin cytoskeleton and regulates cell survival, differentiation and proliferation.^16^, ^17^, ^18^ Besides, it participates in the bidirectional signal transduction process through direct binding or functional combination with intracellular adaptor proteins, cytoplasmic tyrosine kinases and growth factor/cytokine receptors, further affecting cell behaviour.^19^ Therefore, it is reasonable for us to speculate that, during pulmonary development, integrin of airway epithelial cells could be the bridge of the signal transmission between cells and extracellular matrix, by which it performs important functions. Integrins are a family of heterodimeric cell surface receptors which consist of α and β subunits. There is no relative research on the relationship between ITGB4 and lung development.

In this study, we generated epithelial cell‐specific ITGB4‐deleted mice to observe the impact of ITGB4 combined with transcriptome sequencing to testify our hypothesis that ITGB4 could affect lung development.

## 2 METHODS

### 2.1 Mouse strains and animal treatment

All animal experimental studies were approved by the Institutional Animal Care and Use Committee of Central South University (No. 2019‐S102). Referring to the documented establishment of CCSP‐rtTA^tg/−^/TetO‐Cre^tg/tg^ mice expressing Cre recombinase under the control of the club cell secretory protein (CCSP) promoter on a C57BL/6 background,^20^ ITGB4^fl/fl^ mice were bred with CCSP‐rtTA^tg/−^/TetO‐Cre^tg/tg^ mice to generate the CCSP‐rtTA^tg/−^/TetO‐Cre^tg/−^/ITGB4^fl/fl^ triple transgenic mice.

To specifically delete ITGB4 from the airway epithelial cells of CCSP‐rtTA ^tg/−^/TetO‐Cre^tg/−^/ITGB4^fl/fl^ mice, doxycycline (Dox; 0.125 g/300 mL in drinking water) was administered orally from embryonic day 7.5 (E7.5) till postnatal day 42 (P42). ITGB4^fl/fl^ littermates lacking either CCSP‐rt TA, TetO‐Cre, or both were used as controls (β4^f/f^) which were given identical dosage of doxycycline.

### 2.2 ITGB4 siRNA synthesis and transfection

The design and synthesis of ITGB4 siRNA and nonsense siRNA were executed by Guangzhou RiboBio (RiboBio Inc.). Transfections were performed with Lipofectamine 3000 (Invitrogen) according to the manufacturer's instructions.

ITGB4 siRNA: 5′‐CAGAAGAUGUGGAUGAGUU‐3′.

nonsense siRNA: 5′‐UUCUCCGAACGUGUCACGU‐3′.

### 2.3 Cell cultures and treatment

Incubated human bronchial epithelial (HBE) cells, purchased from Lifeline Cell Technology, under the condition of 95% humidity, 37°C with 95% air and 5% carbon dioxide, and changed the medium every 24 h, the collection was performed when cells grew to 95%–100% confluence. Cells were seeded in a 6‐well plate at a density of 2 ~ 5 × 10^5^ cells/well and grown to 50%–70% confluence, subsequently, transfected with siRNA for 48 h, then treated each group with 2.5 μM GSK3β agonist (wortmannin)^21^ (S2758, Selleck. cn), or equal concentrations of DMSO (Sigma‐Aldrich) considered as control.

### 2.4 Bronchoalveolar lavage

Bronchoalveolar lavage fluid (BALF) was collected by the lavaging of the left lungs three times with 0.5 mL of PBS each. Lavage fluid was centrifuged at 750g/min at 4°C, then the total cell counts were performed. Differential blood counts: a smear of the cell pellet of the BALF was prepared, and then examined by Wright‐Giemsa staining (Sigma).

### 2.5 Transcriptome sequencing

Lung tissues were collected at E13.5, P2, P7 and P28 for transcriptome sequencing **(**PRJNA781075**)**. Samples were divided into two groups: β4^ccsp.cre^ mice and the littermate β4^f/f^ mice (considered as control). Total amounts and the integrity of RNA were assessed using the RNA Nano 6000 Assay Kit of the Bioanalyzer 2100 system (Agilent Technologies). Library preparation and clustering for transcriptome sequencing of all the samples were performed by Novogene.

### 2.6 Fetal mice lung explant culture

The E12.5 mouse lung tissues were isolated and placed onto 24‐mm clear polyester membrane supports (Transwell, 0.4 μm pore size; Corning) to create an air–liquid interface, then cultured overnight at 37°C to allow its adherence to the filter. Culture medium (DMEM) was added only to the basal compartment.^22^, ^23^ PBS/DOX was added to the β4^f/f^ group, and PBS/DOX/DOX + wortmannin was added to the β4^ccsp.cre^ group. After being cultured for 48 h, images taken by Motic BA410EF‐UPR microscope were used for newly generated saccular airway branches counting, as documented.^22^

### 2.7 Morphological analysis

Haematoxylin–eosin staining was performed for assessment of lung morphometry, using a 40× objective for six sections per mouse, with a minimum of six mice in each group. Using Image‐Pro Plus software, the mean linear intercept (MLI) and mean septal wall thickness were measured after staining with haematoxylin–eosin.^24^ A scanning electron microscope (SEM) was used for morphology observation of ciliated cells. Mice airways were fixed with glutaraldehyde (2.5%) solution for scanning electron microscopy detection at the College of Life Sciences, Hunan Normal University, Changsha, China.

### 2.8 Statistics

All experiments were independently repeated three times. Data are shown as mean ± SD and were analysed by SPSS19.0 statistical analysis (IBM), visualized by GraphPad Prism software (Version 7.0). Differences between the two groups were determined by a two‐tailed unpaired Student's *t*‐test, while that of multiple groups were tested using analysis of variance (anova) or two‐way anova. For both statistical analyses, *p* < 0.05 was considered as statistically significant.

## 3 RESULTS

### 3.1 Establishment of ITGB4 conditionally knockout mice

**FIGURE 1:**
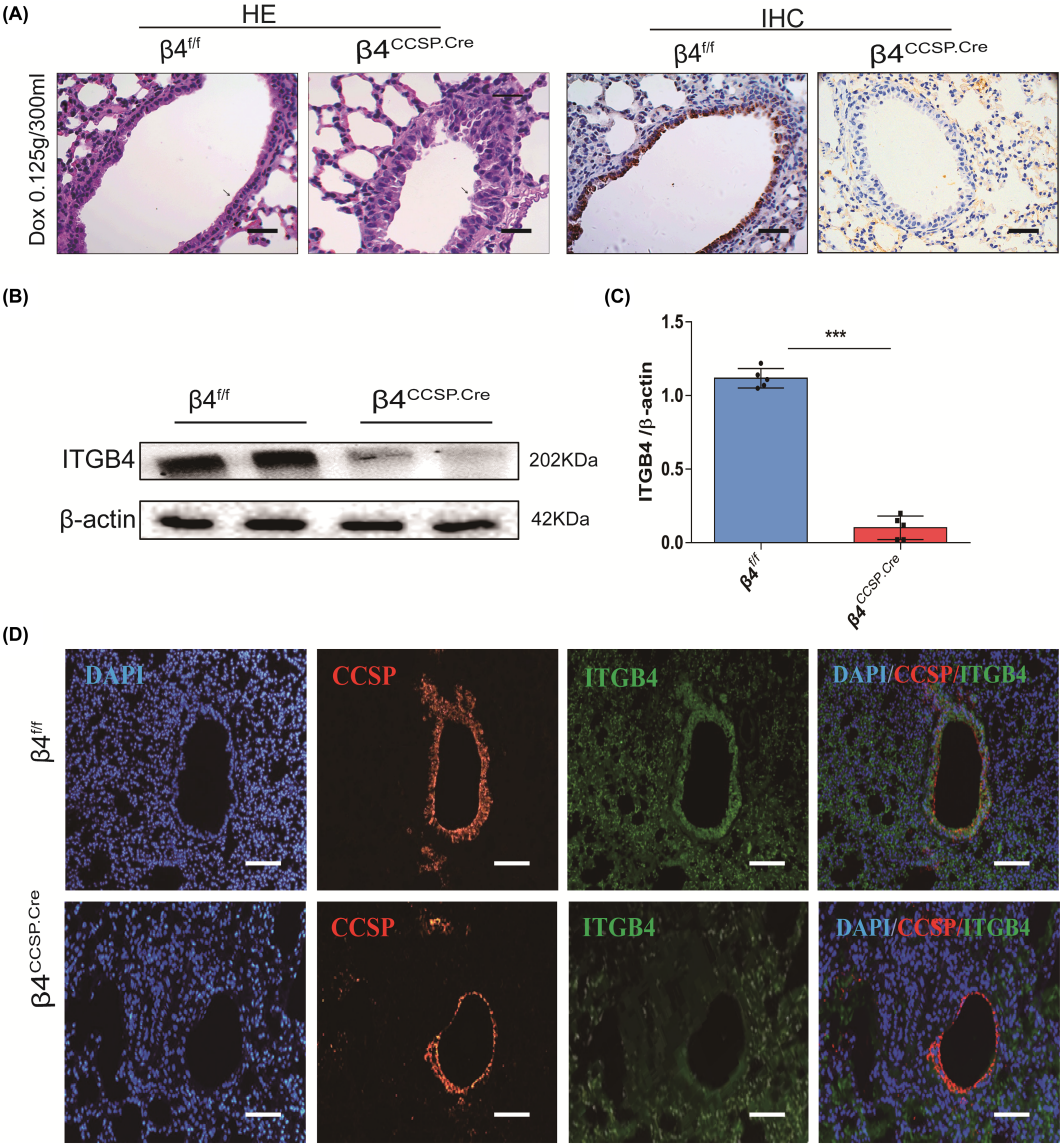
Detection of ITGB4 expression in airway epithelial cells of β4^ccsp.cre^ and β4^f/f^ mice. (A) Detection of ITGB4 expression in airway epithelial cells of β4^ccsp.cre^ and β4^f/f^ mice with Dox of 0.125 g/300 mL with haematoxylin–eosin staining, representative airway epithelial image is shown (×400 magnification; scale bar, 25 μm). (B) ITGB4 protein expression from CCSP^+^ airway epithelial cells was detected by western blot. (C) Quantification WB analysis ***p < 0.001. (D) ITGB4 deficiency validation via immunofluorescence. Co‐localization of CCSP and ITGB4 was performed in lung sections. CCSP and ITGB4 were stained with red and green fluorescent separately. DAPI was used to stain cell nuclei (blue) (×200 magnification; scale bar, 50 μm).

Club cell is a cuboidal, non‐ciliated cell in human and rabbit terminal bronchioles. CCSP is located mainly in the airways. Differentiated lung club cells are marked by the expression of club secretory protein (Cc10/Ccsp/Scgb1a1).^25^ To investigate the role of ITGB4 in lung development, CCSP‐rtTA, Tet‐O‐Cre, or ITGB4^fl/fl^ triple transgenic mice were generated as mentioned in Section 2. Cre‐meditated targeting excision was triggered by doxycycline through oral administration. It has been documented that doxycycline‐inducible Cre recombinase has been targeted to club cells via the CCSP promoter.^26^ Doxycycline is a semisynthetic tetracycline, commonly used for gene conditional knockout. However, a high dosage of Dox could be toxic to organs,^27^, ^28^, ^29^ while the knockout efficiency might be affected by applying a low dosage. Graphical analysis of staining suggested 0.125 g/300 mL as the final concentration, which successfully knockout ITGB4 without affecting the structure of airway epithelium (Figure S1A,B). Further verification of ITGB4 knockout in airway epithelial cells was accomplished by WB and triple immunofluorescence staining. ITGB4 expressed in near‐linear basilar stained airway cells throughout the conducting airways of β4^f/f^ mice, as regards β4^ccsp.cre^ mice, ITGB4 expression was deleted in the conducting bronchi and proximal bronchioles (Figure 1A,D). Western blot analysis of primary CCSP^+^airway epithelial cells also showed that ITGB4 expression was eliminated after Dox treatment (Figure 1B,C).

### 3.2 Airway epithelial‐specific deletion of ITGB4 contributes to lung developmental defects

**FIGURE 2:**
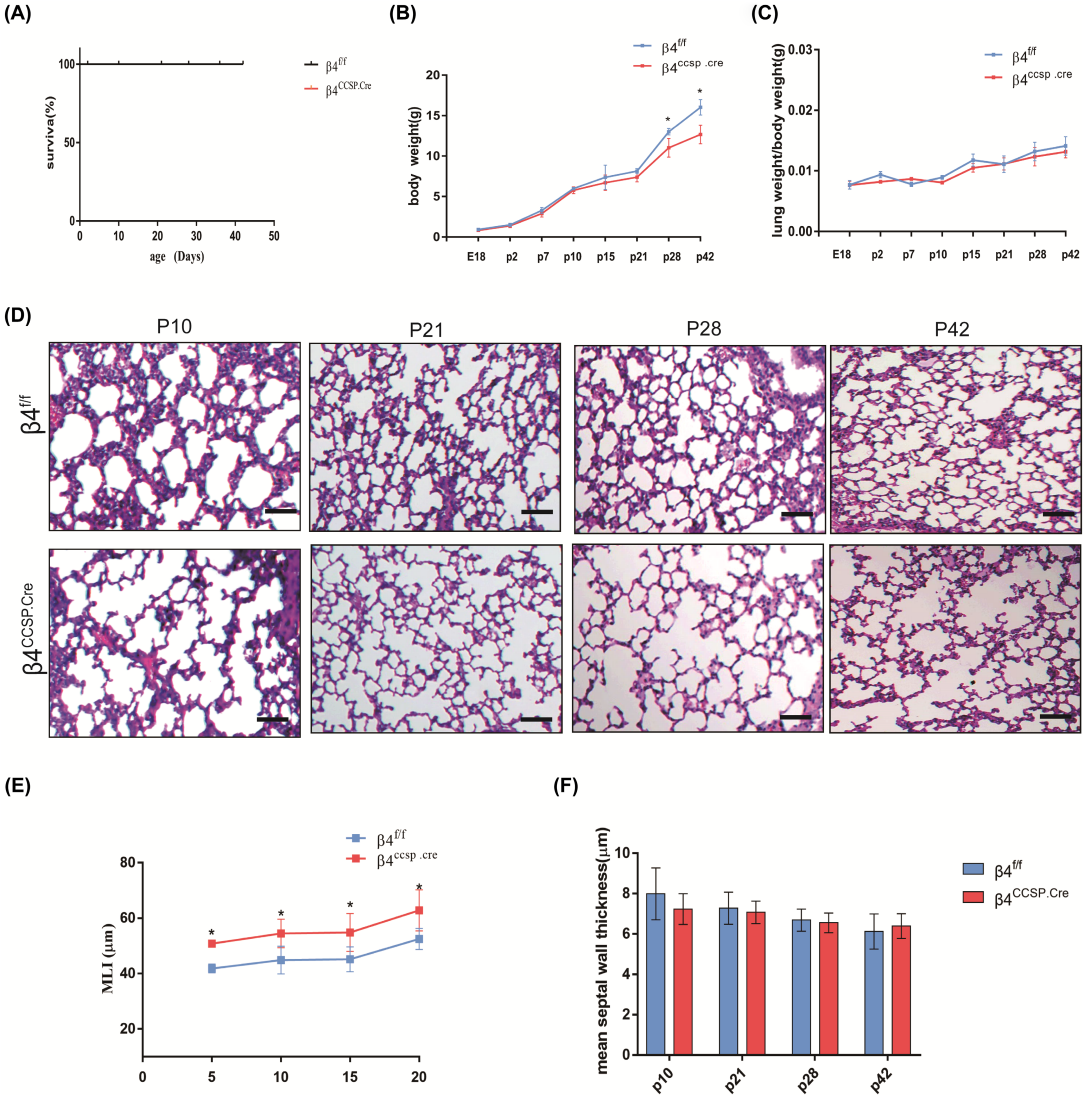
ITGB4 deficiency induced abnormal lung structure without affecting overall survival rates. (A) Kaplan–Meier survival curve for β4^ccsp.cre^ and β4^f/f^ mice (*n* = 15 mice/group) (B) Growth curves for β4^ccsp.cre^ and β4^f/f^ mice (*n* = 15 mice/group). (C) LW/BW curves for β4^ccsp.cre^ and β4^f/f^ mice (*n* = 15 mice/group) (*p < 0.05). (D) Haematoxylin–eosin‐stained paraffin sections of P10, P21, P28 and P42 mice lungs. (*n* = 6 mice/group; ×100 magnification; scale bar, 100 μm). (E) The mean linear intercept is greater in β4^ccsp.cre^ versus β4^f/f^ mice (*n* = 6 mice/group, 10 sections per mouse). MLI of P10, P21, P28 and P42 mice (*p < 0.05). (F) Quantification of alveolar septa at P10, P21, P28 and P42.

**FIGURE 3:**
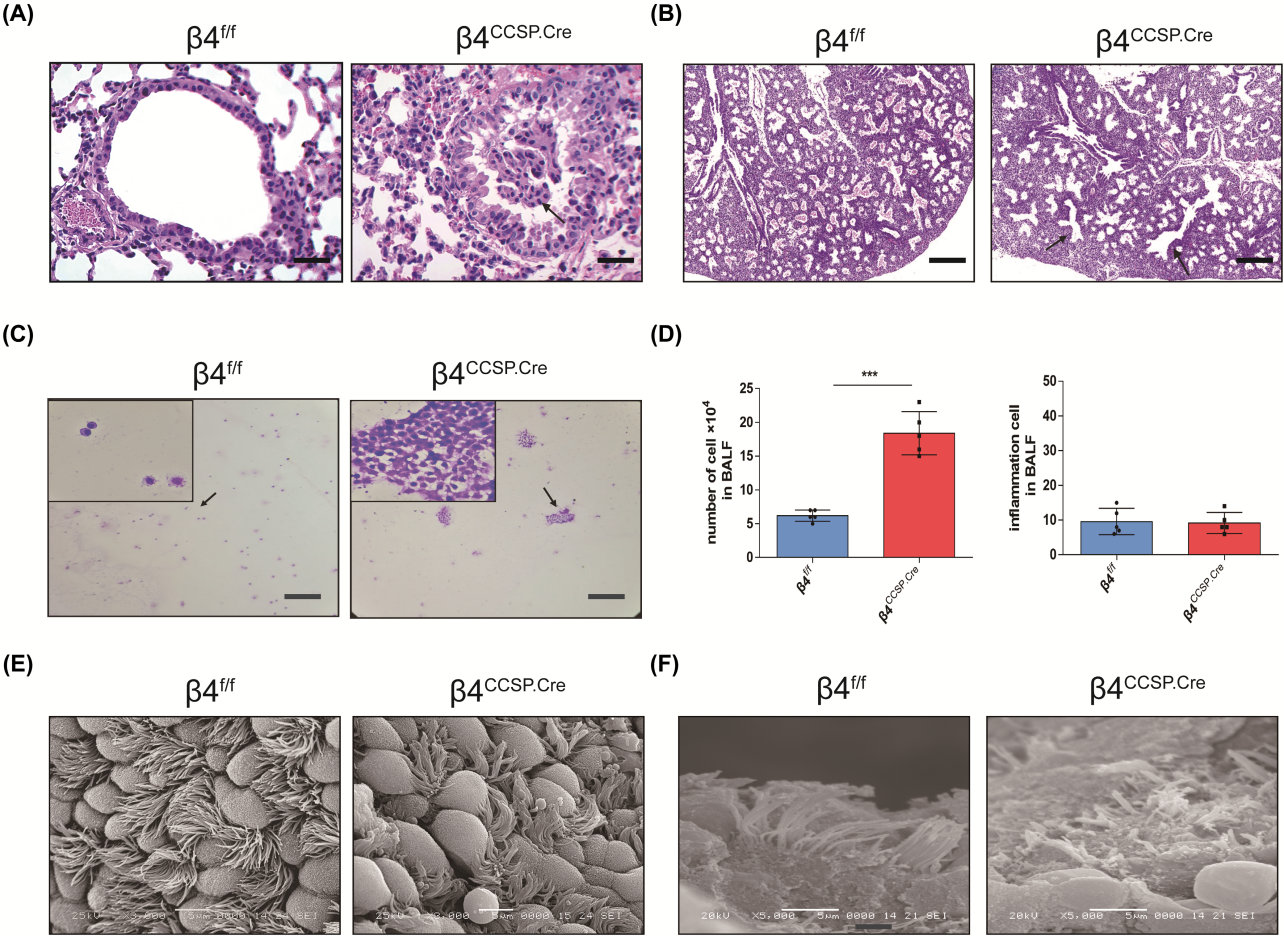
Airway epithelial‐specific deletion of ITGB4 contribute to branch, epithelium and cilia defects. (A) Haematoxylin–eosin‐stained paraffin sections of P28 β4^f/f^ and β4^ccsp.cre^ mice for the observation of epithelium impairments (×400 magnification, scale bar, 25 μm). (B) Haematoxylin–eosin‐stained paraffin sections of β4^f/f^ and β4^ccsp.cre^ lungs at E18. Decreased branching and the number of airspaces per mm^2^ are present in E18 β4^ccsp.cre^ lungs compared with β4^f/f^ lungs (×100 magnification; scale bar, 100 μm). (C) Denudated airway epithelium in the BALF (×100 magnification, scale bar, 100 μm). (D) Cell counting in the BALF (*n* = 5; ***p < 0.001). (E, F) Scanning electron microscopy (SEM) showing micrographs of the epithelium of the trachea in β4^ccsp.cre^ and β4^f/f^ mice. The data represent the cumulative results of six independent experiments. The airway epithelial cilia of the β4^ccsp.cre^ group appeared to be lodging (E), reduced or shortened (F) (*n* = 5; E: ×3000 magnification; F: ×5000 magnification; scale bar, 5 μm).

We observed the physiological and morphological features of mice at different development stages. There was no significant difference in body weight between β4^ccsp.cre^ mice and age‐matched littermate control β4^f/f^ at birth; however, β4^ccsp.cre^ mice became significantly lighter than age‐matched β4^f/f^ mice 4 weeks after birth, and the gap widened as they grew. (Figure 2B). But the overall survival rates and the ratio of lung weight to body weight (LW/BW) were not significantly impacted by ITGB4 deficiency (Figure 2A,C). Lungs harvested at various developmental stages were analysed by haematoxylin–eosin and SEM. Although no structural difference was observed at E13.5 between β4^ccsp.cre^ and β4^f/f^ mice, visible defects in branching morphogenesis emerged at E18. Transverse or coronal lung sections from β4^ccsp.cre^ embryos showed fewer but more dilated airway tubules (Figure 3B). These results showed that β4 deficiency had no significant effect on the overall development of mice. Abnormal lung development occurred early in the development of mice and the weight difference was observed at 4 weeks after birth, which indicated that the later weight difference of mice was secondary.

Histological examination of lung parenchyma harvested at various time points from P10 to P42 showed that the lung structures of β4^ccsp.cre^ mice manifested typical characteristics of alveolar simplification, as indicated by enlarged alveoli with decreased secondary septa, irregular alveolar shape with an emphysema‐like enlargement of distal airspaces (Figure 2D,E). The same result was reported in another paper of our group.^7^ Nevertheless, no obvious thickening of the alveolar septum was found (Figure 2F). The airway epithelium of β4^ccsp.cre^ mice appeared to have maturation arrest and epithelial disorder, represented by the abnormal structure of epithelial cells. Besides, the damaged epithelium was prone to denudate and slough into the airway lumen, which could be observed in both lung tissue (Figure 3A) and alveolar lavage fluid (Figure 3C). Impairment of cilial growth or structure was observed using SEM, showing that the airway epithelial cilia of β4^ccsp.cre^ group appeared to be reduced, shortened, lodging and sparse, and large areas of non‐ciliated airway epithelium emerged (Figure 3E,F).

The above results indicated that ITGB4 defect in the early stage of lung development might lead to BPD.

### 3.3 Effect of ITGB4 deficiency on development‐relevant factors

To further investigate the effect of ITGTB4 deletion on lung development, we detected the expression of relevant factors.

**FIGURE 4:**
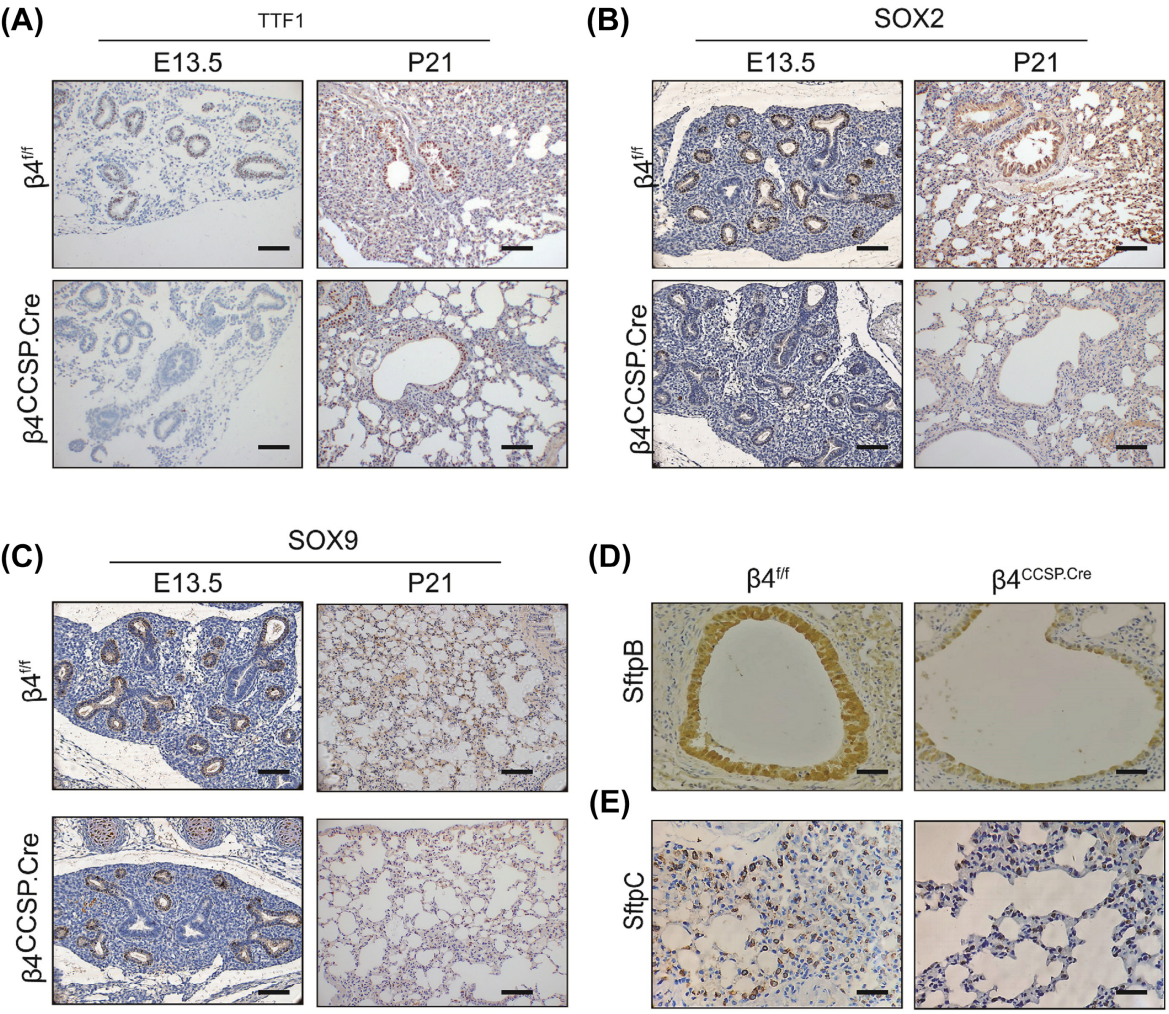
Effect of ITGB4 defects in airway epithelial on development‐related molecules. Immunohistochemical staining of TTF1 (A), SOX2 (B) and SOX9 (C) in the airway epithelium at E13.5, P21 (×200 magnification, scale bar, 50 μm). Immunohistochemical staining of sftpB (D) and sftpC (E) in the airway epithelium at P42 (×400 magnification, scale bar, 25 μm).

TTF1 is the earliest molecule marker of lung development, regulating branching morphogenesis and the secretion of surfactant protein.^30^ TTF1 protein expression was examined in multiple stages of development from E13.5 to P21 by IHC. Compared with the control group, the expression of TTF1 in the β4^ccsp.cre^ group was markedly downregulated in the pseudoglandular stage, which is the primary branching morphogenesis stage, and in the alveolar stage (Figure 4A). Besides, the expression of surfactant protein B(SftpB) (Figure 4D) and SftpC (Figure 4E), the marker of Type II alveolar epithelial cells, was also significantly decreased, which suggested that conditionally knocking out ITGB4 from airway epithelium in the early stage of lung development can not only impede alveolar epithelial cells differentiating into alveolar epithelial Type II cells but also reduce the secretion of pulmonary surfactant protein. Proximal and distal epithelial progenitor cells are labelled with SOX2 and SOX9. IHC showed that SOX2 expression is significantly reduced induced by ITGB4 defect in the primary branching morphogenesis stage and the alveolar stage (Figure 4B), while the expression of SOX9 remained unchanged (Figure 4C). It showed that the specific knockout of ITGB4 in the embryonic stage has a significant effect on the development of the proximal lung; however, the unaltered expression of SOX9 suggested that SOX9 might not be involved in the impairment of distal lung development. However, no significant changes were observed in the mRNA and protein levels of TGFβ1, SHH, and FGF10 and other growth factors ((Figure S2). These results suggest that β4 integrin deficiency may not regulate the progression of BPD by regulating the expression of these growth factors.

Lung inflammation is also essential to bronchopulmonary dysplasia (BPD).^31^ To investigate whether inflammation was involved in the development of BPD, we carried out differential blood counts in BALF. And the counts of β4^ccsp.cre^ mice group were significantly bigger than the control group, though they were mainly composed of detached airway epithelial cells, while the counts of inflammatory cells showed no difference, which was consistent with our observation from haematoxylin–eosin staining (Figure 3D). We also carried out macrophage clearance assay, in which clodronate liposomes were used to deplete macrophage, and mice treated with PBS‐loaded liposomes were used as control. The result showed that abnormal structure of airway epithelial cells and alveolar cavity enlargement of similar degree appeared in the lung tissues of both groups treated with clodronate liposomes or PBS‐loaded liposomes (Figure S3A,B). It indicated that the BPD observed in β4^ccsp.cre^ mice might be because of the deficiency of ITGB4.

### 3.4 Transcriptome of airway epithelial‐specific deletion of ITGB4

To further explore the reason why ITGB4 deficiency induced BPD, transcriptome sequencing was performed on four different developmental stages: pseudoglandular stage (E13.5), saccular stage (P2), alveolar stage I (P7), alveolar stage II (P28).^14^, ^32^, ^33^

**FIGURE 5:**
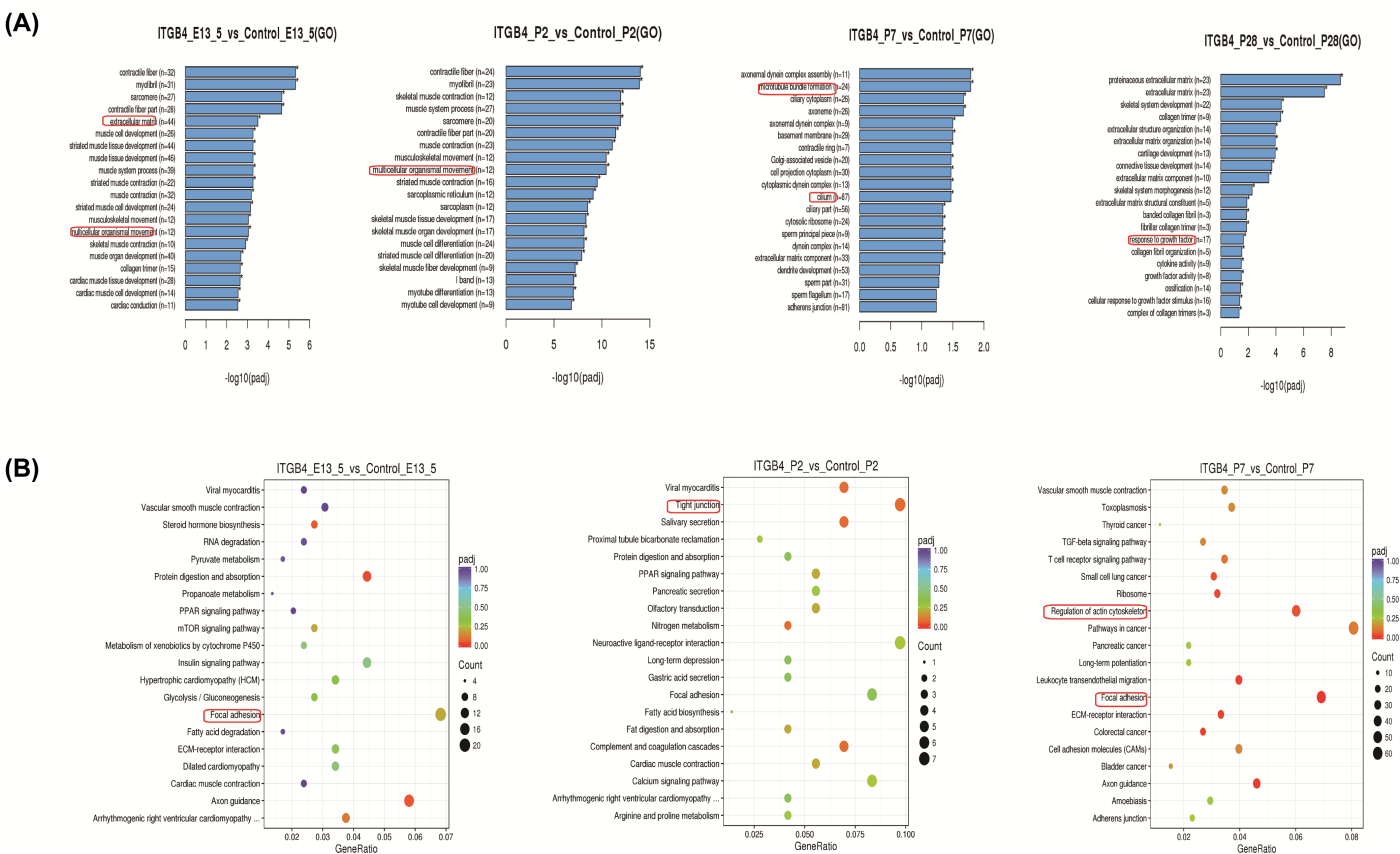
Transcriptome sequencing of lung tissue from ITGB4‐deficient mice and wild‐type mice. (A) GO functional enrichment analysis in β4^f/f^ and β4^ccsp.cre^ group at E13.5, E2, P7 and P28. (B) A bubble chart shows the top 20 enriched GO terms. The size of the dot represents the proportion of genes, which is positively associated with the proportion of corresponding enrichment items. The change in colour from dark blue to red represents a change in *p*‐value from high to low.

Differently expressed genes (DEGs) between the β4^f/f^ and β4^ccsp.cre^ groups were screened out by high‐throughput transcriptome sequencing in each paired group of all 4 stages, combining GO and KEGG pathway enrichment analysis. DEGs were mainly enriched in the extracellular matrix and multicellular organismal movement at the pseudoglandular stage. At the alveolar stage I, DEGs were mainly enriched in the cilium, microtubule bundle formation. As for alveolar stage II, DEGs were mainly enriched in growth factor activity and cellular response to growth factor stimulus (Figure 5A). During the four stages of lung development, KEGG enrichment analyses suggested that most differentially expressed genes were involved in focal adhesion, regulation of actin cytoskeleton, etc (Figure 5B).

### 3.5 ITGB4 might regulate lung development through FAK/GSK3β/SOX2 signal pathway

**FIGURE 6:**
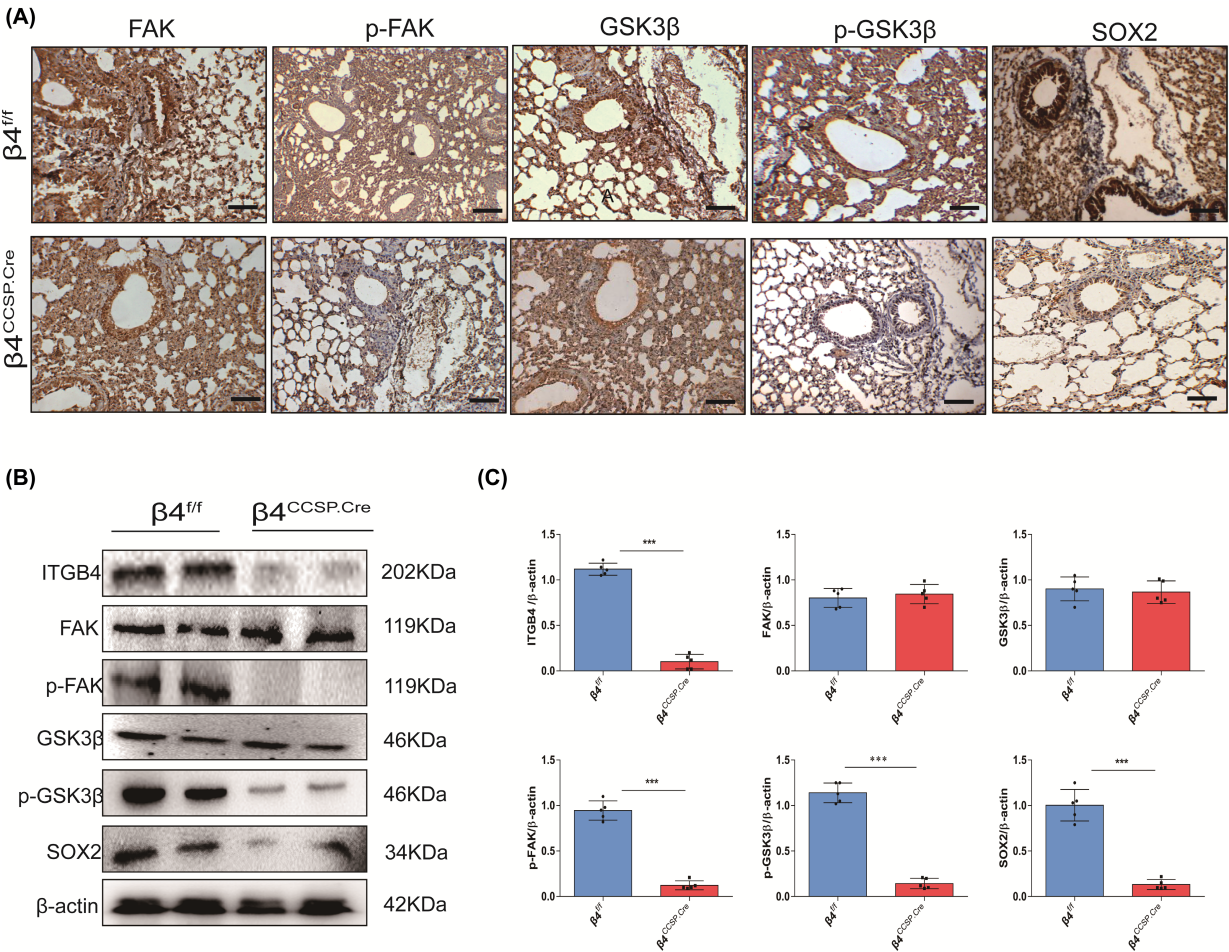
Effects of ITGB4 deletion on FAK/GSK3β/SOX2 pathway in mice. (A) Immunohistochemical staining of FAK, phospho‐FAK, GSK3β, phospho‐GSK3β and SOX2 at P28. (B) Western blotting detection of the expression of ITGB4, FAk, phospho‐FAK, GSK3β, phospho‐GSK3β and SOX2 at P28. (C) Quantification of western blotting (*n* = 5; ***p < 0.001).

An accumulation of literature has reported that the focal adhesion kinase (FAK) is located at the intersection of multiple signal transmission pathways, involving cell migration, adhesion and other physiological activities, and is important for mediation of signalling downstream of integrins and growth factor receptors.^34^ The results of transcriptomics and protein level showed that FAK expression did not change significantly after β4 deletion; however, the expression of phospho‐FAK decreased significantly (Figure 6A). It suggests that β4 integrin deficiency activated the FAK pathway. Additionally, SOX2 gene, which is the downstream of the FAK signalling pathways, was downregulated in the preceding results (Figure 4B). Glycogen synthase kinase 3β(GSK3β) is also the downstream molecule of FAK. It is reported that GSK3β could be regulated by FAK and itself could regulate SOX2 through its phosphorylation.^35^ Thus, we speculated that FAK/GSK3β/SOX2 could be the underlying mechanism of BPD induced by ITGB4 conditional knockout.

**FIGURE 7:**
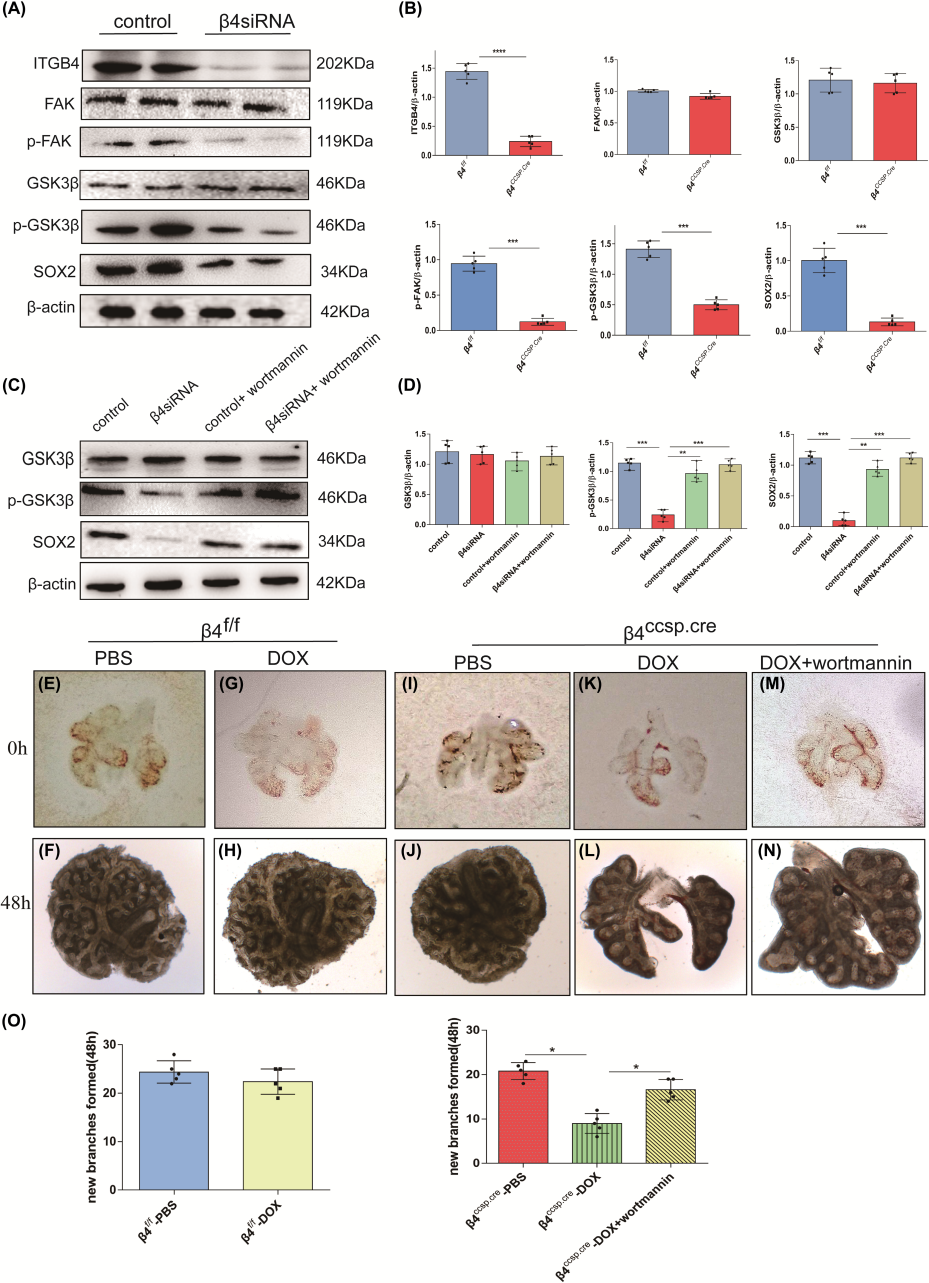
ITGB4 influences lung development through the FAK/GSK3β/SOX2 signal pathway. Silencing ITGB4 by small interfering RNA (siRNA) transfection in HBE cells. Experimental groups were treated with 25 μM wortmannin. The control group was prepared with equivalent concentrations of DMSO. (A, C) ITGB4, FAK, phospho‐FAK, GSK3β, phospho‐GSK3β and SOX2 expression was detected by western blotting. (B, D) Quantification of western blotting (*n* = 5; *p < 0.05; **p < 0.01; ***p < 0.001). (E–H) Culture of fetal lung explants from E12 β4^f/f^ mice. Dox was used for the elimination of its own effect on branching morphogenesis. (I–N) Culture of fetal lung explants from E12 β4^ccsp.cre^ mice showing decreased branches compared with control, and recovery after wortmannin treatment. (O) Quantification of the newly generated branches (*n* = 5; *p < 0.05).

To prove our hypothesis, we detected the expression of molecules related to the FAK/GSK3β/SOX2 pathway. The results indicated no significant changes in the expression of FAK and GSK3β upon loss of β4 expression. But the decreased expression of phospho‐FAK, phospho‐GSK3β and SOX2 is obviously detected (Figure 6A–C). The experiments performed on HBE agreed with the results mentioned above (Figure 7A,B). Correspondingly, protein levels of phospho‐FAK, phospho‐GSK3β and SOX2 recovered after being treated with wortmannin (Figure 7C,D). Fetal lung explant culture was carried out to analyse the effects of ITGB4 on branching morphogenesis. The addition of Dox did not affect branching morphogenesis. Compared with controls, significantly less new branching occurred in β4^ccsp.cre^ lung explants after cultured for 48 h (Figure 7E–H), which was consistent with previous results (Figure 3B). Branching morphogenesis was evaluated by cultures of fetal lung explants treated with or without wortmannin (Figure 7E–O). Although wortmannin treatment did not fully rescue airway branching defects (Figure 7M,N), partial recovery was achieved compared with the control group without wortmannin treatment (Figure 7K,L). Taken together, these data indicate that deficiency of β4 integrin disrupts lung development through FAK/GSK3β/SOX2 signal pathway.

## 4 DISCUSSION

Epithelial–mesenchymal interactions play an essential role in the developing lungs and in adult lungs.^36^ Integrins are transmembrane proteins and surface adhesion receptors; they mediate the interaction of cells and the extracellular matrix, which is vital for the formation, maintenance and repair of tissues as well as for other biological processes such as the metastasis of cancer cells. ITGB4 has a unique structure and subcellular localization, making it the most unusual molecule of all the β integrin subunits, the cytoplasmic domain of which is bigger than other β subunits, including a juxtamembrane domain and two pairs of Type III Fn‐like repeats.^37^, ^38^, ^39^ Deleting ITGB4 in airway epithelial cells with 0.125 g/300 mL DOX at E7.5 resulted in abnormal lung development and weight loss, but the overall survival rate was not affected, which indicated that ITGB4 defect was not lethal. It has been published that ITGB4 participated in mammary gland development and maintain the integrity of mammary architecture, ITGB4 disruption impedes branching morphogenesis in immortalized normal breast cell.^40^ In our research, a variety of ITGB4 knockout‐induced lung development defects were observed at multiple time points, including altered branching morphogenesis, impaired alveolarization, differentiation defects of epithelial cells and cilia disorders, indicating that ITGB4 expressed in airway epithelial cells has multiple functions in different stages of lung development, and it is reasonable for us to speculate that these manifestations could exist during the whole lifespan of β4^ccsp.cre^ mice.

RNA and protein levels showed that ITGB4 deficiency did not affect the expression of common growth factors, and the transcriptome results indicated that most DEGs were mainly enriched in extracellular matrix, focal adhesion and regulation of actin cytoskeleton. FAK, a tyrosine kinase with a molecular weight of 125kD, is an essential member of integrin‐mediated signal transduction located at the intersection of multiple signal transmission pathways.^41^, ^42^ FAK phosphorylation can be induced through the intracellular domain of integrin β subunit and then through src (sarcoma gene), phosphatidylinositol‐3‐kinase (PI3K) and other pathways to introduce extracellular matrix signals into the cell, activating downstream gene expression, actin polymerization, cell migration and a series of biological activities. It has been reported that the mutation of FAK phosphorylation site Y397F can reduce the expression of phosphorylated FAK and effectively inhibit FAK‐mediated cell migration.^43^ The cytosolic domain of ITGB4 includes a variety of tyrosine phosphorylation sites, and a large number of cytoskeletal and signaling proteins have been known to bind to these sites located in the tail of the cytosolic domain. ITGB4 influences cell signaling by modulating cell signaling pathways involving transmembrane protein kinases such as FAK and tyrosine receptor kinases. Maintaining normal cell motility requires dynamic regulation of adhesion signals.^24^, ^44^ The serine/threonine protein kinase GSK3β is a highly conserved protein kinase, which regulates many physiological pathways by phosphorylating key downstream targets.^45^ GSK3β can be regulated by FAK, and its phosphorylation can also regulate its downstream molecule SOX2.^35^ SOX2 is of great significance for the differentiation of proximal progenitors into various progeny, whose defects result in the loss of the mature secretory and ciliated lineages of lung airways.^46^, ^47^ Another paper of our group found that migration and proliferation of ITGB4 deficiency cells were noticeably inhibited, along with decreased cytoskeleton stabilization.^7^ In this study, we observed that despite the fact that no significant changes of FAK and its downstream molecule, GS3Kβ, were observed, the expression of phospho‐FAK and phospho‐GSK3β were downregulated in β4‐silenced HBE and lung tissues sampled from β4‐defect mice compared to each control group. Treatment with wortmannin restored the expression of phospho‐GSK3β and SOX2, in addition, although branching defect still existed, it was partially rescued. This suggests that ITGB4 can regulate the expression of downstream molecules by downregulating the phospho‐FAK and then inhibiting the phospho‐GSk3β. The expression of SOX2 was significantly downregulated in both ITGB4 deficient lung tissue and β4‐silenced cell line. Meanwhile, the secretion of SftpB and SftpC was reduced; besides, there were also manifestations such as lodged and shortened cilia, whose number was decreased too, its impact could sustain till adulthood. Thus, we inferred that the impairments of lung development induced by ITGB4 knockout could be realized through FAK/GSK3β/SOX2 pathway.

It has been reported that deleting β1 integrin in lung epithelium beginning at E10.5 results in abnormal lung development with a 100% fatality rate at the age of 16 weeks.^12^ Based on our observation, knockout of ITGB4 in airway epithelial cells did not affect the overall survival rate; besides, in our previous research, we observed that after LPS treatment, the mRNA expression of anti‐inflammatory factors, including IL‐10 and ARGA‐1, was significantly reduced in the lung tissue of β4^ccsp.cre^ mice compared with wild‐type mice.^48^ Under the stimulation of HDM, it is more likely for β4^ccsp.cre^ mice to acquire airway hyper‐responsiveness and asthma than wild‐type mice.^39^ These results suggest a different role of β4 than β1 in lung development. Though it is not lethal, during lung developmental stages, ITGB4 defects might affect multiple signaling pathways and the resistance of fetal lung to the environment changes inside and outside the uterus, leading to pulmonary diseases and being associated with many other pulmonary dysplasia‐related diseases. Furthermore, it has a long‐term effect on pulmonary function, making it more susceptible to acquired chronic respiratory system diseases.

In our research, we attribute the defects of lung development to the impacts ITGB4 has on FAK/GSK3β/SOX2 pathway; however, ITGB4 itself is a crucial factor of epithelial–mesenchymal interactions, and the execution of its function could involve multiple signaling pathways. Thus, its impact might not only be restricted to lung development but could also be related to other pulmonary diseases. Therefore, research on fetal lung development mechanisms can not only pave the way for the exploration of therapeutic targets of neonatal lung diseases but also have important guiding significance for the prevention and treatment of adult congenital or acquired lung diseases.

## AUTHOR CONTRIBUTIONS

**Yu Chen:** Data curation (lead); formal analysis (lead); investigation (equal); methodology (lead); software (equal); writing – original draft (lead); writing – review and editing (lead). **Wang Jiang:** Data curation (supporting); investigation (equal); methodology (equal). **Jin‐Mei Wang:** Data curation (supporting); investigation (supporting). **Xiao‐Di Ma:** Formal analysis (supporting); software (supporting). **Di Wu:** Visualization (supporting). **Le‐Xin Liu:** Visualization (supporting). **Ming Ji:** Conceptualization (supporting). **Xiang‐Ping Qu:** Project administration (supporting). **Chi Liu:** Conceptualization (supporting). **Hui‐Jun Liu:** Validation (supporting). **Xiao‐Qun Qin:** Writing – review and editing (supporting). **Yang Xiang:** Funding acquisition (lead); project administration (equal).

## FUNDING INFORMATION

This work was supported by the National Natural Science Foundation of China (grant numbers 81670002, 81970033 and 82070034).

## CONFLICT OF INTEREST STATEMENT

The authors confirm that there are no conflicts of interest.

## CONSENT FOR PUBLICATION

The authors listed have approved the manuscript that is enclosed.

## Supporting information


Data S1:

## Data Availability

All data generated or analysed during this study are included in this published article (the data that support the findings of this study are available from the corresponding author upon reasonable request).
