# Supplementary material for: Conditional knockout of ITGB4 in bronchial epithelial cells directs bronchopulmonary dysplasia
Source: J Cell Mol Med. 2023 Sep 12;27(23):3760–72. doi: 10.1111/jcmm.17948 (PMC10718146; doi:10.1111/jcmm.17948)
Supplement: Supplementary file 1 — Data S1: [file JCMM-27-3760-s001.docx]

**Supplementary figure**


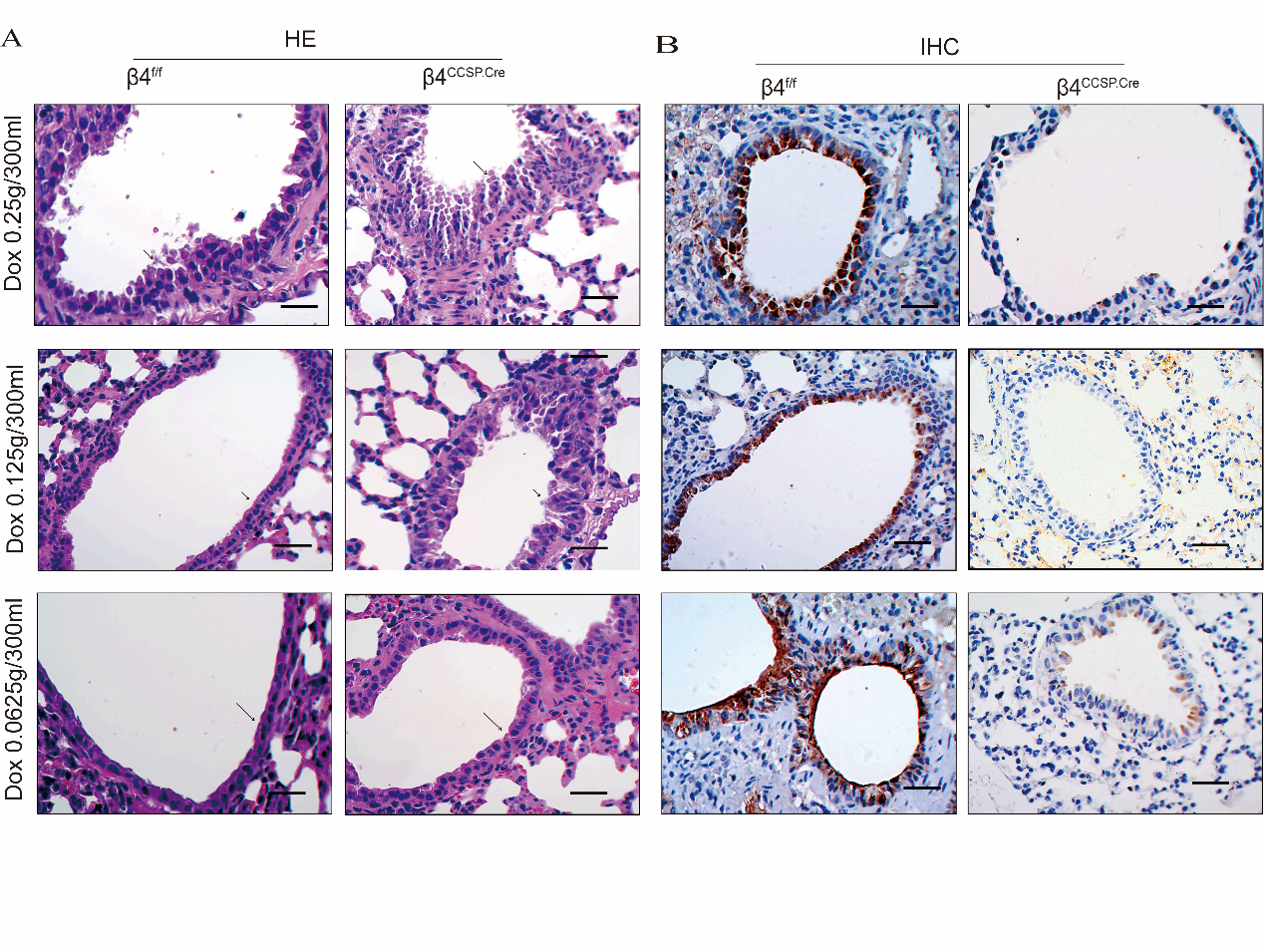


**Fig. S1 Screening of Appropriate doxycycline concentration**

(A)Doxycycline concentration was screened with immunohistochemical (B)and H&E staining(A), representative airway epithelial image is shown (×400 magnification; scale bar, 25µm).

Concentration gradient was built for the screening, the effect of different concentrations of doxycycline on lung morphology and knockout efficiency was evaluated respectively by HE and IHC staining. At the concentration of 0.25g/300mL, ITGB4 was successfully knocked out, but we also observed the impairment and detachment of epithelium in both β4^CCSP.Cre^and β4^f/f^ mice, indicating the toxic effects of DOX of 0.25g/300 mL to cells. such phenomenon was eliminated after treatment with Dox of 0.0625g/300 mL, but ITGB4 was not completely knocked out. Graphical analysis of staining suggested 0.125g/300mL as the final concentration which successfully knockout ITGB4 without affecting the structure of airway epithelium


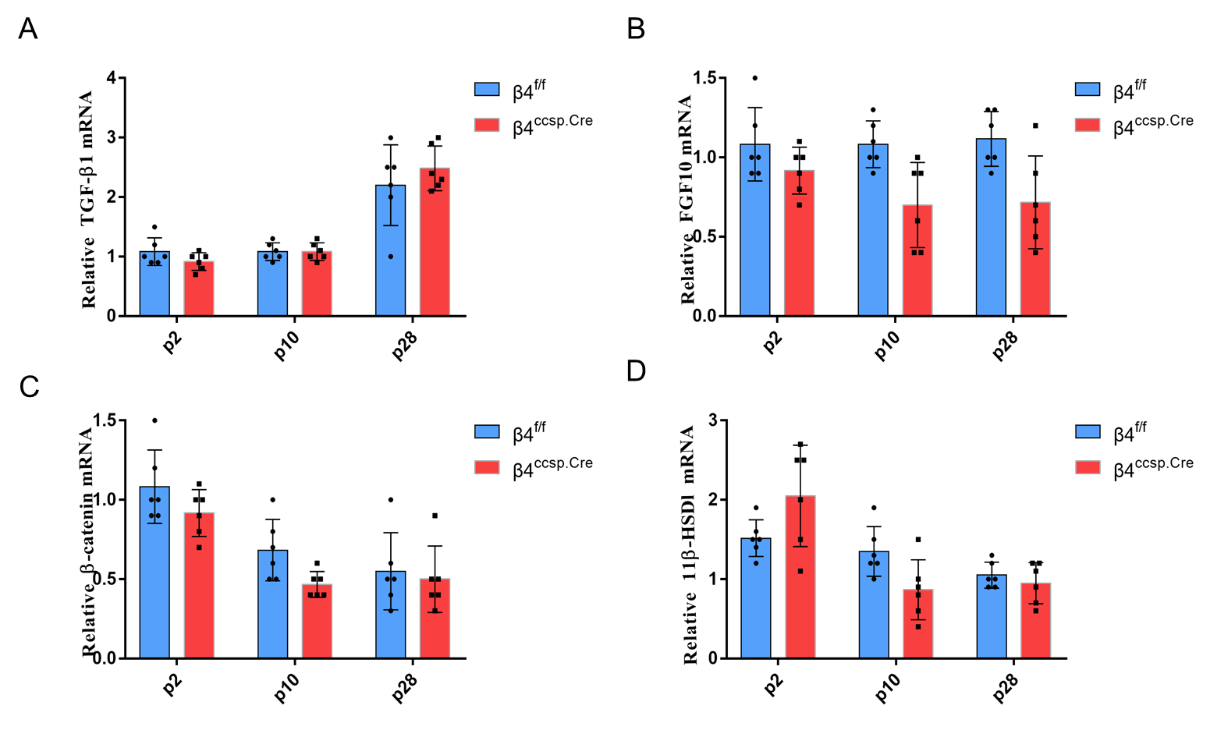


**
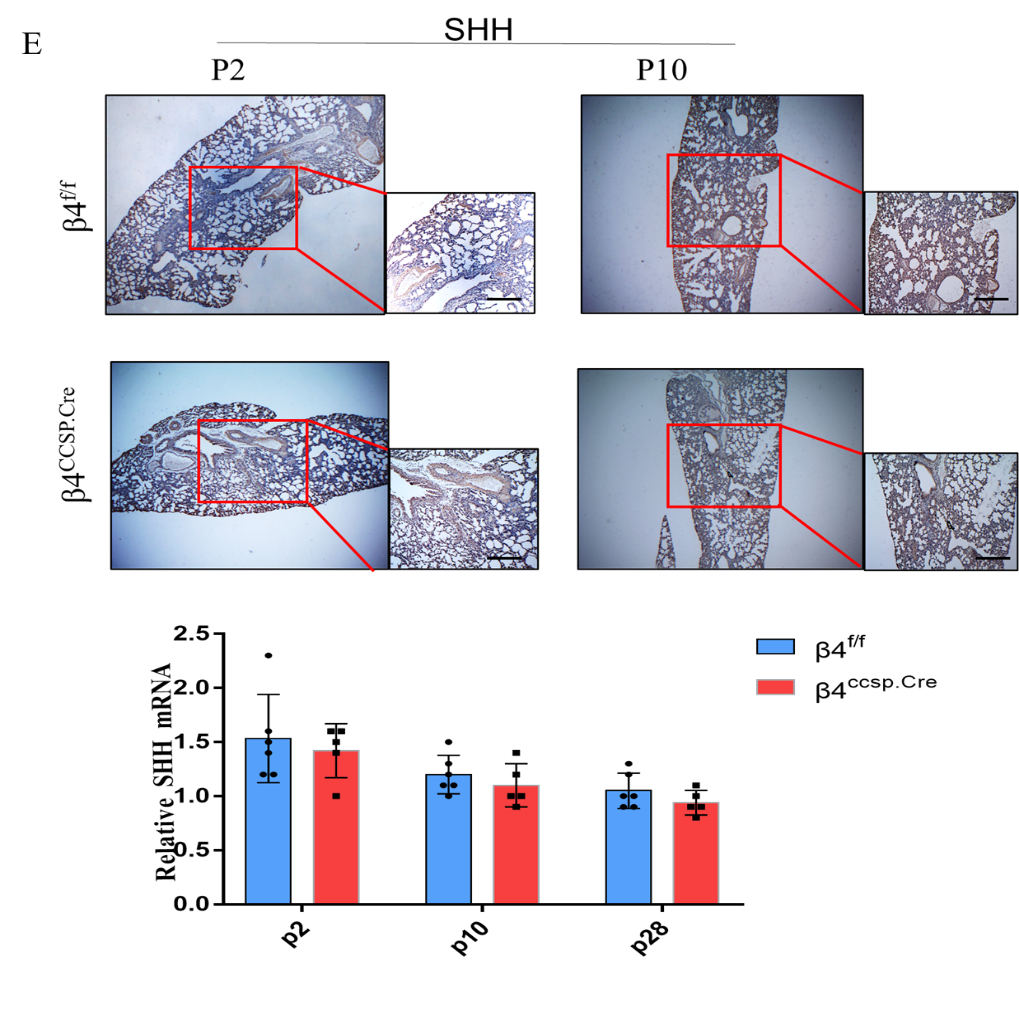
**

**
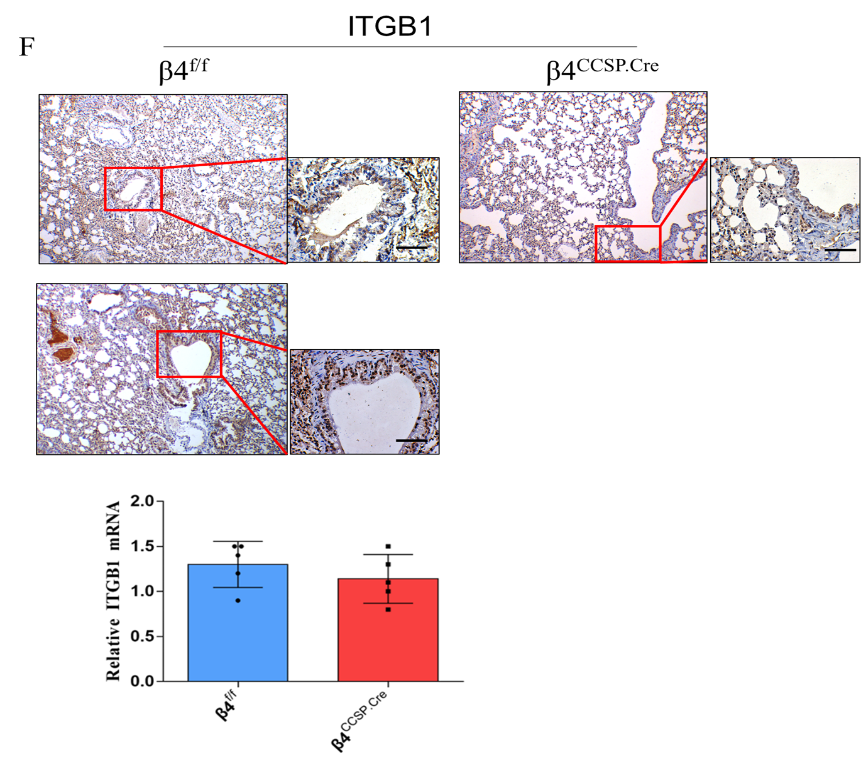
**

**Fig. S2 No effect of ITGB4 defect on the expression of a part of the development-related molecules.**

The mRNA expression of TGFβ1(A), FGF10(B), β-catenin(C)，11β-HSD1(D); (E)The expression of SHH at p2 and p10 as shown by IHC staining (×100 magnification, scale bar, 100µm) and the mRNA expression of SHH; (F) The expression of ITGB1 as shown by IHC staining (×400 magnification, scale bar, 25µm) and the mRNA expression of ITGB1.


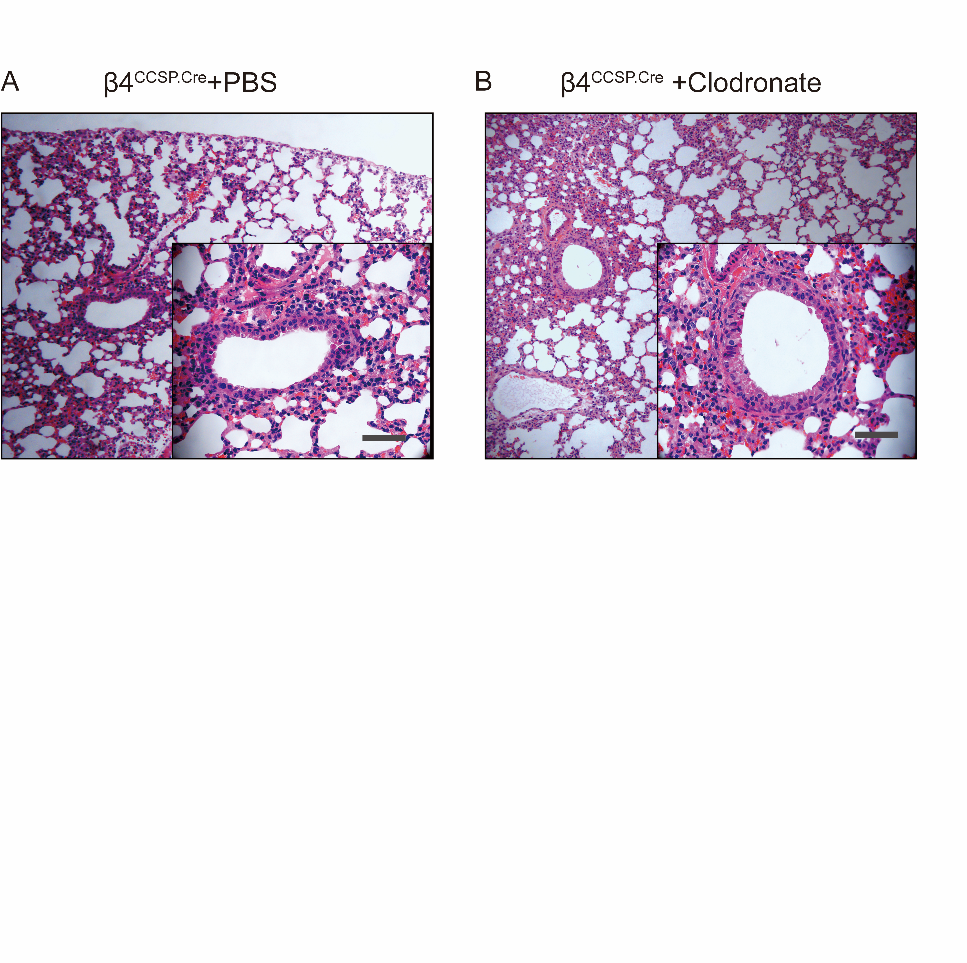


**Fig. S3** **Macrophage clearance assay**

multiple layers of airway epithelium and enlarged alveolar airspaces appear both in the clodronate liposomes treated group(A)and PBS-loaded liposomes treated group(B). (×400 magnification, scale bar, 25µm)

**Supplementary Methods**

**Western blotting**

Total protein extraction was performed using the mixture containing RIPA and protease inhibitor (100:1), and quantified by the BCA kit. Centrifuged the lysates at the condition of 12,000 rpm for 20min, 4°C, and took the supernatant for detection. Separated by 10% SDS-PAGE gels, the supernatant was then transferred to a PVDF membranes, blocked with 5% non–fat milk for 2 hours at room temperature on a shaking table.

Then, ITGB4 primary antibody (dilution 1:1000; ab182120, Abcam), SOX2(dilution 1:6000, GTX101507, GeneTex), FAK (dilution 1:1000, 40913, SAB), phospho-FAK (dilution 1:1000, 13327, SAB), GSK3β (dilution 1:1000, BF8003, Affinity), phospho-GSK3β (dilution 1:1000, AF2016, Affinity), and β-actin (dilution 1:5000; ab, Abcam) were added and incubated at 4°C overnight. The following day, washed the membrane in TBST for 3 times, 5 mins each. Subsequently, incubated in the secondary antibody (1:5000 dilution) at room temperature for 30 minutes and quantified the relative band intensity using the Image Lab Analyzer software (Bio‐Rad, Hercules, CA).

**Immunostaining analysis**

Lung lobes were fixed in 4% paraformaldehyde and embedded with paraffin. The expression of ITGB4 and SOX2 were detected by both immunofluorescent (IF) and immunohistochemical staining (IHC). Expression of other relative factors was detected by immunohistochemical staining. Antibodies used: CCSP (sc365992,Santa Cruz), ITGB4(ab182120,Abcam)，TTF1(ab76013, Abcam), SOX2(GTX101507, GeneTex), SOX9(ab185966, Abcam), FAK(40913,SAB), p-FAK(13327, SAB), GSK3β(BF8003, Affinity), phospho-GSK3β(AF2016, Affinity), sftp B(ABS21, Merck Millipore), sftp C(ab211326, Abcam).

**Macrophage clearance assay**

Macrophage clearance assay was conducted with clodronate (dichloromethylene diphosphonic acid; Sigma-Aldrich) and sterile PBS-containing liposomes (vehicle) as previously described(Sinha et al., 2018; Zaynagetdinov et al., 2011). β4^CCSP.Cre^ mice were treated with intranasal clodronate or PBS-containing liposome vehicle every 5 days beginning at P5 until P14 or P28 for harvest. Different doses of intranasal clodronate were given respectively at P5, P10, P15, P20 and P25: 12μl, 15μl, 18μl, 21μl and 24μl. PBS-containing liposome vehicle control were used for duplicate experiments.
